# Association Mapping for Aluminum Tolerance in a Core Collection of Rice Landraces

**DOI:** 10.3389/fpls.2016.01415

**Published:** 2016-10-04

**Authors:** Peng Zhang, Kaizhen Zhong, Hanhua Tong, Muhammad Qasim Shahid, Jinquan Li

**Affiliations:** ^1^State Key Laboratory of Rice Biology, China National Rice Research InstituteHangzhou, China; ^2^State Key Laboratory for Conservation and Utilization of Subtropical Agro-Bioresources, South China Agricultural UniversityGuangzhou, China; ^3^Department of Plant Breeding and Genetics, Max Planck Institute for Plant Breeding ResearchCologne, Germany

**Keywords:** Ting’s core collection, rice aluminum tolerance, relative root elongation, association mapping, mixed linear model, allele effect

## Abstract

Trivalent aluminum (Al^3+^) has drastic effect on the rice production in acidic soils. Elite genes for aluminum (Al) tolerance might exist in rice landraces. Therefore, the purpose of this research is to mine the elite genes within rice landraces. Association mapping for Al tolerance traits [i.e., relative root elongation (RRE)] was performed by using a core collection of 150 accessions of rice landraces (i.e., Ting’s rice core collection). Our results showed that the Ting’s rice core collection possessed a wide-range of phenotypic variation for Al tolerance, and the index of Al tolerance (RRE) was ranged from 0.22 to 0.89. Moreover, the groups with different origins and compositions of *indica* and *japonica* rice showed different degrees of tolerance to varying levels of Al. These rice landraces were further screened with 274 simple sequence repeat markers, and association mapping was performed using a mixed linear model approach. The mapping results showed that a total of 23 significant (*P* < 0.05) trait–marker associations were detected for Al tolerance. Of these, three associations (13%) were identical to the quantitative trait loci reported previously, and other 20 associations were reported for the first time in this study. The proportion of phenotypic variance (*R*^2^) explained by 23 significant associations ranged from 5.03 to 20.03% for Al tolerance. We detected several elite alleles for Al tolerance based on multiple comparisons of allelic effects, which could be used to develop Al tolerant rice cultivars through marker-assisted breeding.

## Introduction

Acidic soils occupy approximately 30% of the world’s ice-free area, and over 50% of the world’s arable land is acidic (Vonuexkull and Mutert, 1995; Kochian et al., 2004; Ryan et al., 2011). Aluminum (Al) is the most abundant metal in the earth’s crust. Al is solubilized as trivalent Al (Al^3+^) in acidic soils with pH values below 5.0. Al has been shown to be beneficial to some plant species including rice when supplied at low concentrations, and Al’s benefits were summarized in a review of Pilon-Smits et al. (2009). Though beneficial at low level, Al^3+^ at high concentrations inhibits root growth, damages root systems, and causes significant reductions in crop yields (Liu et al., 2012). Al^3+^ is the major toxic cation encountered by plants on acidic soils (Delhaize et al., 2012b). Up to 60% of the acidic soils in the world occur in developing countries, where food production is affected by Al toxicity in acid land and always a major area of research for plant physiologists and breeders (Kochian et al., 2005; Delhaize et al., 2012a; Fujii et al., 2012; Xia et al., 2013; Caniato et al., 2014; Li et al., 2014). Therefore, identification of elite genes for Al tolerance is of paramount importance for plant growth and production in the world.

Rice (*Oryza sativa* L.) is the most Al tolerant crop among small grain cereals (Ma et al., 2002) and is one of the world’s most important crops, supplying food for nearly half of the world’s population, especially in Asia. About 13% of global rice produced on acidic soils (Vonuexkull and Mutert, 1995). Therefore, improving Al tolerance of rice is highly valuable for rice production. In recent years, many scientists have studied genetic mechanism of Al tolerance in rice (Huang et al., 2009; Yamaji et al., 2009; Xia et al., 2010; Famoso et al., 2011; Li et al., 2014; Arenhart et al., 2016; Yokosho et al., 2016). Most of the rice researches were focused on Al tolerance at seedling stage, and reported some rice accessions or mutants which were tolerant or sensitive to Al by measuring relative root elongation (RRE). Furthermore, previous studies illuminated that there were two detoxification mechanisms under Al^3+^ threaten, i.e., exclusion of Al from root cells by producing and excreting chelating chemicals (Yokosho et al., 2011) and accumulation of Al and internal detoxification (Huang et al., 2009; Yamaji et al., 2009; Arenhart et al., 2013, 2014).

A number of quantitative trait loci (QTLs) for Al tolerance have been identified in rice by using different mapping populations (Nguyen et al., 2002; Ma and Furukawa, 2003; Mao et al., 2004; Xue et al., 2007; Famoso et al., 2011), and a few genes conferring Al tolerance have been identified (Huang et al., 2009, 2012; Yamaji et al., 2009; Yokosho et al., 2011; Chen et al., 2012; Xia et al., 2013; Li et al., 2014). However, QTL mapping was done by using conventional linkage mapping methods in the segregating populations derived from crossing between typical Al tolerant and sensitive rice varieties in the previous studies. The linkage mapping have major limitations, including only two alleles at any given locus can be studied in bi-parental crosses, high cost and poor mapping resolution (Flint-Garcia et al., 2003), whereas association mapping could overcome the limitations of linkage mapping (Kraakman et al., 2004) and enables researchers to use modern genetic technologies to exploit natural genetic diversity and identify elite genes in the genome (Zhu et al., 2008). However, as far as we know, association mapping by using a natural population was seldom performed in the previous studies on Al tolerance.

An appropriate population with maximized phenotypic variation is critical for the success of an association analysis (Yan et al., 2011). Rice landraces represent an intermediate stage in domestication between wild and elite cultivars (Londo et al., 2006), which possess high genetic diversity and many exotic genes, and thereby provide useful germplasm resources for rice breeding. Moreover, association mapping based on a core collection of rice landraces would help to attain as much phenotypic variation as possible. However, the knowledge about the association mapping in a core collection of rice landraces for the Al tolerance trait is rather limited. Though Famoso et al. (2011) have performed association mapping for Al tolerance by using 383 diverse rice accessions, they were not complete rice landraces. Moreover, these rice accessions were not from a complete core collection.

As one of the earliest rice collections in China, Ting’s rice collection was collected by the researcher Ying Ting during 1920–1964 from all over China as well as from Korea, Japan, Philippines, Brazil, Indonesia, Australia, and Vietnam (Li and Zhang, 2012). The original collection comprises of 7128 rice landraces. In our previous studies, a core collection with 150 accessions was constructed from 2262 accessions out of 7128 based on a strategy of stepwise clustering and preferred sampling on adjusted Euclidean distances and weighted pair-group average method using integrated qualitative and quantitative traits (Li et al., 2011). Population structure and linkage disequilibrium (LD) level of the rice core collection had been examined in details (Zhang et al., 2011). A large number of accessions in Ting’s core collection showed distinct Al tolerance. Moreover, the Ting’s core collection has been used for association mapping of agronomic traits, and some of the mapping results with the core collection could be confirmed in other two mapping populations as well as being consistent with previous mapping results (Zhang et al., 2014). Therefore, the Ting’s core collection could be an appropriate population for association mapping studies of Al tolerance.

In the present study, an association mapping for AL tolerance was carried out using the Ting’s core collection of rice landraces with 274 simple sequence repeat (SSR) markers. The study aimed to (1) identify phenotypic variations for Al tolerance in the rice landraces of the core collection, (2) perform association mapping for Al tolerance using the core collection, and (3) identify potential alleles of the loci that showed significant trait–marker associations with Al tolerance. These information will be very useful for the rice breeders to enhance the resistance of elite cultivars against Al.

## Materials and Methods

### Plant Material

The Ting’s core collection with 150 accessions of rice landraces (Li et al., 2011) was used in this study. The information for these accessions is shown in Supplementary Table S1. In addition, two Al tolerant varieties, i.e., Nipponbare and Xiangnuo 1 (Yang et al., 2007) as well as three Al sensitive varieties, i.e., Nante (Fu et al., 2010), Xiangzhongxian 2 (Xu et al., 2004), and IR64 (Khatiwada et al., 1996) were selected to identify the most accurate concentration of Al toxicity in this study.

### Screening for Al Tolerance

Uniform seeds of the two Al tolerant and three Al sensitive varieties were surface sterilized in 1% H_2_O_2_ for 30 min, and then washed with deionized water for several times. After this, rice seeds were soaked to germinate in deionized water overnight at 30°C for 2 days in dark. The seedlings were transferred to a plastic net floating on a 0.5 mmol l^-1^ CaCl_2_ (pH = 4.0) solution in a 1.5-l plastic container, and seedlings were grown at 28°C. The solution was renewed daily and no other nutrient solution was provided. Previous researches indicated that a simple CaCl_2_ solution method could be used to screen Al tolerance for very young seedlings when the seed is still capable of providing all necessary mineral nutrients and avoid the problem of Al precipitation in Yoshida’s solution (Famoso et al., 2010; Xia et al., 2010, 2013; Yokosho et al., 2011, 2016). Therefore, the simple CaCl_2_ solution method was applied in this study. After 48 h, the seedlings for Al toxicity treatment were exposed to 0.5 mmol l^-1^ CaCl_2_ (pH = 4.0) containing 50, 100, 150, 200, 250, 300, 350, 400, 450, and 500 μmol l^-1^ AlCl_3_ (no other nutrient solution was applied), while the seedlings for control were exposed to 0.5 mmol l^-1^ CaCl_2_ (pH = 4.0) only. Then RRE was used to evaluate the degrees of Al tolerance of all varieties. RRE was calculated by following formula: RRE = root length under Al toxicity treatment/root length without Al toxicity treatment. The root length of 10 seedlings of each variety was measured with a ruler before and after the treatments (24 h) in one replicate, and six replications were performed. Then the most accurate Al^3+^ concentration for the detection of Al tolerance was selected, and that Al^3+^ concentration was further used to measure the Al tolerance of Ting’ core collection.

All of rice seeds of the 150 varieties from Ting’s core collection were harvested from the farm of South China Agricultural University, Guangzhou (23°16N, 113°8E), during the late season (July–November). Uniform seeds and seedlings were treated as aforementioned method with the determined Al^3+^ concentration from the previous step. Similarly, the root length of 10 seedlings of each accession was measured with a ruler before and after the treatment (24 h) in one replicate, and six replications were performed. Then the RRE value for each accessions of the Ting’s core collection was calculated to measure Al tolerance. In the present study, RRE ≥ 0.5 was used as a criterion to find out Al tolerant varieties (Fu et al., 2010).

### Genotyping

A total of 274 SSR markers, evenly distributed across the 12 chromosomes of rice, were selected to genotype all varieties of Ting’s core collection (Supplementary Table S2). A total of 23, 25, 24, 22, 21, 22, 21, 25, 23, 24, 23, and 21 markers were mapped to chromosomes 1–12, respectively. The average distance between the loci in chromosomes 1–12 was 7.5, 8.2, 9.4, 7.4, 7.1, 6.3, 5.8, 5.4, 5.2, 4.7, 5.6, and 5.3 cM, respectively. Markers with prefix RM were selected from previously published data (Chen et al., 1997; Temnykh et al., 2000; McCouch et al., 2002), and those with prefix position-specific marker (PSM) were taken from the thesis of Huang (2003). DNA was extracted using a modified sodium dodecyl sulfate (SDS) method (Zheng et al., 1995). The volume of the polymerase chain reaction (PCR) was 10 μl. The profile of the PCR program was as follow: 94°C for 5 min followed by 29 cycles of 94°C for 1 min, 55°C for 1 min, and 72°C for 1 min with a final extension of 5 min at 72°C. PCR products were separated by electrophoresis on a 6% polyacrylamide gel and detected by silver staining (Panaud et al., 1996). A standard marker (100–600 bp, produced by Shanghai Biocolor BioScience & Technology Company) was added on each gel as a control. The size of PCR products were detected by BIO Imagine System with software Genetools from SynGene and were manually re-checked twice to reduce the errors (Zhang et al., 2011). The length of each allele was compared to the bands of the standard marker for scoring.

### Data Analysis

Means and standard deviation (SD) for root length and RRE were calculated using Excel software. The percentage of phenotypic variation explained by population structure was calculated using a general linear model with software SPSS 17.0 for Windows (SPSS Inc. Chicago, IL, USA). The broad-sense heritability (H^2^) was calculated as $\begin{array}{c} H^{2}=\sigma_{g}^{2}/\left(\sigma_{g}^{2}+\sigma_{e}^{2}\right) \end{array}$ using software QGA Station 1.0^1^, where σ_g_^2^ is the genetic variance, and σ_e_^2^ is the environmental variance. Polymorphism information content, which measures the extent of polymorphism for marker gene(s) or marker sequence(s), was calculated using the program POWERMARKER V3.25. Software Structure V2.3.1 was used to infer the population structure and to get Q matrices (Pritchard et al., 2000a,b). During the running, a range of genetic clusters from K = 1–15 with the admixture model was examined, and five replications were used for the estimation of each K. Each run implemented with a burn-in period of 100,000 steps followed by 100,000 Monte Carlo Markov Chain replicates. An ad hoc measure ΔK was used to detect the numbers of subgroups. That run with the maximum likelihood was applied to subdivide the varieties into different subgroups based on the maximum membership probability. A Q-matrix was obtained from the membership probability of each variety. The Q-matrix was used for further association mapping. The Loiselle algorithm was chosen for calculating the kinship matrix (K) by software SPAGeDi (Hardy and Vekemans, 2002). Association analysis was performed using the software TASSEL^2^. For the mixed linear model (MLM), both K and Q matrices were incorporated. Significance of associations between marker and traits were determined by their P values (P < 0.05), which were calculated by the statistical models, and the phenotypic variance explained by the significant loci was calculated through ANOVA. Rare alleles with the frequency of less than 10% in a population were filtered as missing data in association analysis. Quantile–quantile plots were generated for observed against expected -log_10_ (P) using software SAS version 9.0 (SAS Institute 2002), where observed P values were obtained from the association mapping and expected P values from the assumption that no associations happened between marker and trait.

## Results

### Determination of Al Toxicity Concentration

**FIGURE 1:**
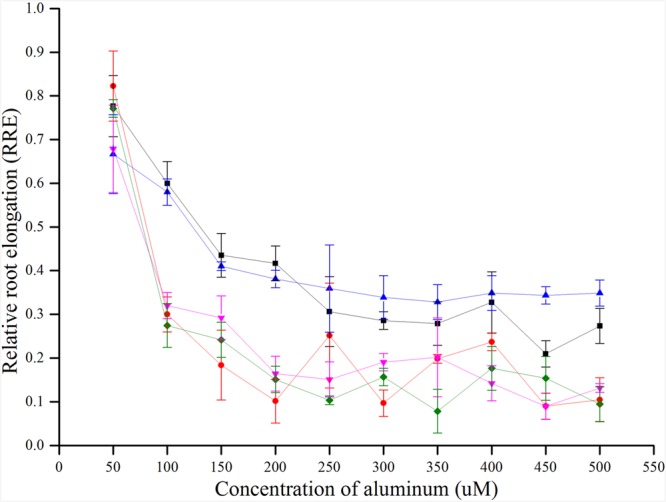
**RRE of five CKs (controls) treated with different concentrations of Al^3+^ for 24 h**.

There was no uniform concentration to measure the Al toxicity in previous studies. In this study, five rice varieties (control varieties, CK) that have been reported as Al tolerant and sensitive were used to determine an appropriate concentration for Al toxicity or tolerance. The RRE of five CKs decreased with the increase in concentration of Al^3+^. When the Al^3+^ concentration was about 300 μmol l^-1^, the RRE reached a plateau and no further remarkable decline was found by increasing the Al^3+^ concentration. Furthermore, the RRE of five CKs decreased sharply when Al^3+^ concentration increased from 50 to 100 μmol l^-1^, and a maximum difference in RRE was observed at 100 μmol l^-1^ for the two tolerant and three sensitive CKs (**Figure 1**). Therefore, 100 μmol l^-1^ was determined as the most accurate concentration for Al toxicity.

### Identification of Al Tolerance Traits

**FIGURE 2:**
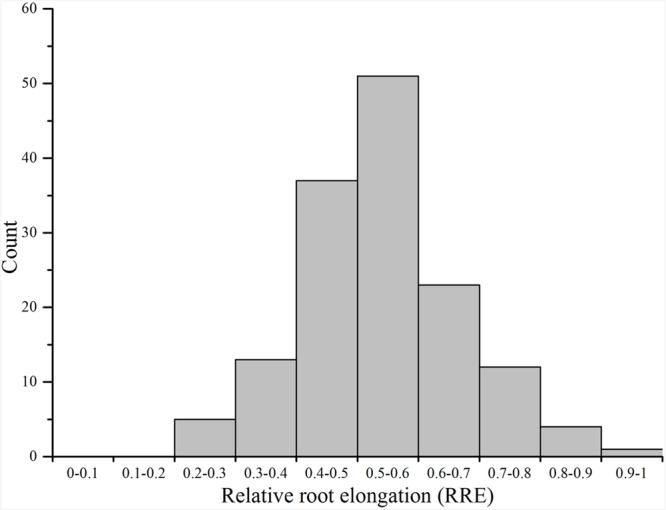
**Distribution of aluminum tolerance in the Ting’s core collection**.

Ting’s core collection showed a wide-range of phenotypic variation for Al tolerance, as indicated by the measurement of RRE, i.e., ranged from 0.22 to 0.89 with an average of 0.54. Moreover, 63.70% of the 150 varieties had larger than 0.50 RRE (**Figure 2**). The variety with the largest RRE was a typical *indica* variety “Chang ning wu qu nan tou zhan,” while the variety “Ai you” with the smallest RRE belong to typical *japonica* subspecies. The broad-sense heritability for Al tolerance was 88.73% (**Table 1**).

**Table 1:** Distribution of aluminum tolerance in the entire collection, subgroups, and sub-subgroups identified by STRUCTURE.

Group	*N*	Definition	Relative root elongation (RRE) ± SD	Range	The percentage of RRE ≥ 0.5	*h*^2^ (%)	*R*^2^ (%)
Ting’s core collection	150		0.54 ± 0.12	0.22∼0.89	63.70		
SG 1	111		0.52 ± 0.11	0.22∼0.77	52.78		
SG 1a	24	*I*-IS	0.54 ± 0.09	0.33∼0.71	66.67		
SG 1b	34	*I*-ST	0.46 ± 0.11	0.22∼0.73	29.41	88.73	42.00
SG 1c	21	*I*-LS	0.54 ± 0.09	0.37∼0.77	47.62		
SG 1d	32	*I*-ES	0.55 ± 0.10	0.34∼0.75	65.63		
SG 2	21		0.59 ± 0.19	0.29∼0.89	76.19		
AD	18		0.57 ± 0.12	0.30∼0.77	72.22		

**I, Indica*; *J, Japonica*; ES, early season; LS, late season; IS, intermediate season; ST, subtropical. *N* is the sample size. *R*^2^ and *h*^2^ represent the percentage of phenotypic variation explained by population structure and heritability in broad sense, respectively.*

As 42.0% of phenotypic variation of Al tolerance was influenced by population structure (**Table 1**), it is necessary to compare the Al tolerance within subgroups and even sub-subgroups of the core collection. In our previous study, three subgroups were presented in the Ting’s core collection, i.e., SG 1 (*indica*), SG 2 (*japonica*), and AD (admixed) as well as four sub-subgroups were detected in the SG 1, including SG 1a (intermediate season landraces), SG 1b (subtropical landraces), SG 1c (late season landraces), and SG 1d (early season landraces) (Zhang et al., 2011).

**FIGURE 3:**
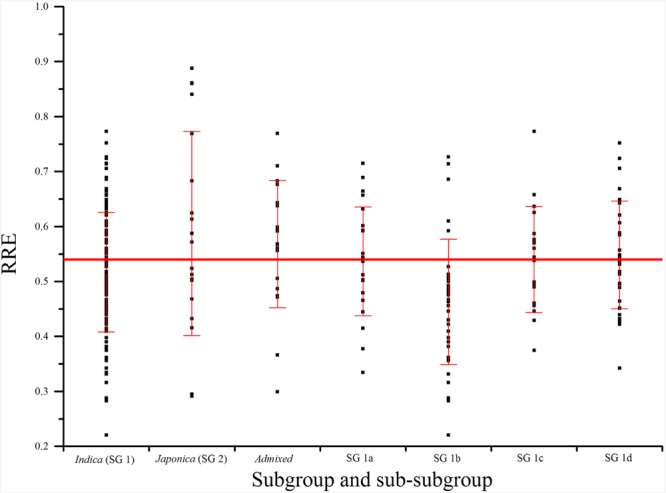
**Phenotypic variations (RRE) for aluminum tolerance among subgroups and sub-subgroups**. The horizontal line indicates mean of RRE in Ting’s core collection. SG 1, SG 2, SG 1a–d, and admixed were identified in our previous studies using software STRUCTURE (Zhang et al., 2011).

In subgroup SG 1, RRE ranged from 0.22 to 0.77 with an average of 0.52, and 52.78% of genotypes exhibited larger than 0.50 RRE. In subgroup SG 2, RRE ranged from 0.29 to 0.89 with an average of 0.59, and 76.19% of landraces showed larger than 0.50 RRE. In AD, RRE ranged from 0.30 to 0.77 with an average of 0.57, and 72.22% of RRE values were larger than 0.50 (**Figure 3**; **Table 1**). In sub-subgroup SG 1a, RRE ranged from 0.33 to 0.71 with an average of 0.54, of these RRE values, 66.67% were larger than 0.50. In sub-subgroup SG 1b, RRE ranged from 0.22 to 0.73 with an average of 0.46, and only 29.41% of RRE values were larger than 0.50. In sub-subgroup SG 1c, RRE ranged from 0.37 to 0.77 with an average of 0.54, among them 47.62% of RRE displayed higher levels than 0.50. In sub-subgroup SG 1d, RRE ranged from 0.34 to 0.75 with an average of 0.55, and 65.63% landraces displayed higher than 0.50 RRE (**Figure 3**; **Table 1**).

### The Effect of Controlling Type I Error Using MLM

**FIGURE 4:**
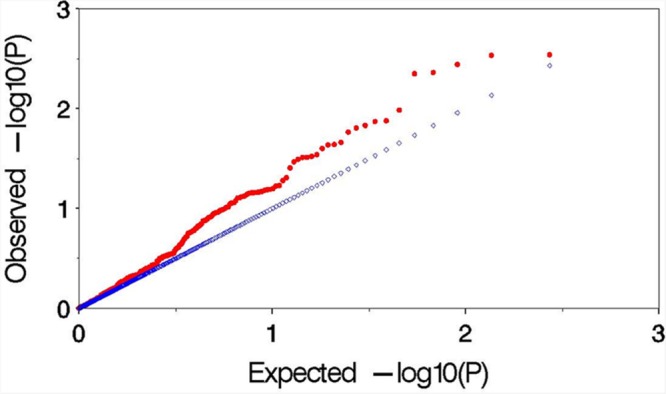
**Plot of observed versus expected *P* values by using MLM (Q+K) model for the association mapping for aluminum tolerance.** The blue and red symbols are representing the expected *P* values and observed *P* values, respectively.

Observed versus expected *P* values for each trait–marker association were plotted to assess the control of type I errors. Uniform distributions between the observed and expected *P* values were observed for all the traits, and demonstrated by similar distributions in 2 years (**Figure 4**). The result indicated that MLM method effectively control the false positives in this study.

### Trait–Marker Associations for Al Tolerance

A total of 23 trait–markers depicted significant associations (*P* < 0.05) with Al tolerance using MLM approach, and a total of 3, 2, 3, 2, 1, 1, 1, 2, 3, 3, 1, and 1 trait–marker associations were mapped to chromosomes 1–12, respectively. Moreover, three (13%) of 23 trait–marker associations for Al tolerance were identical to the previously reported QTLs (**Table 2**), and the other 20 trait–marker associations were new trait–marker associations for Al tolerance. The percentage of the phenotypic variation (*R*^2^) explained by significant associations ranged from 5.03% (for RM341) to 20.03% (for PSM365), with an average of 11.65% (**Table 2**).

**Table 2:** Significant (*P* < 0.05) associations between SSR markers and aluminum tolerance.

No.	Marker names	Chromosome no.	Mb position	*P-*value (MLM)	*R*^2^ (%)	Candidate gene ID	Description	Previous mapped genes or QTLs
1	PSM41	1	2.15	0.0044	16.59	Os01g0140700	Regulator of the ATPase of the vacuolar membrane	None
2	RM220	1	4.42	0.0146	6.3	Os01g0183633	60S ribosomal protein L18a-like protein None	None
3	RM306	1	15.66	0.0321	7.29	None	PAN domain-containing protein At5g03700	None
4	RM341	2	19.34	0.0288	5.03	Os02g0527900	None	None
5	PSM374	2	21.46	0.0043	11.79	None	None	None
6	RM7	3	9.83	0.0134	15.63	None	Hypothetical protein	None
7	PSM377	3	15.39	0.0299	15.07	Os03g0386600	40S ribosomal protein S19	*CQL3* (Wu et al., 2000)
8	RM156	3	17.71	0.017	6.02	Os03g0424500	None	None
9	RM252	4	25.36	0.0029	15.33	None	None	None
10	RM559	4	35.33	0.0036	14.8	None	None	None
11	PSM341	5	0.66	0.0103	12.45	None	protein CURVATURE THYLAKOID 1A, chloroplastic None	None
12	RM136	6	8.75	0.0339	12.94	Os06g0264800	Alpha/beta hydrolase fold-1 domain containing protein	None
13	PSM142	7	3.56	0.0307	18.39	None	None	*QAlRr7.1* (Nguyen et al., 2003)
14	RM407	8	0.52	0.039	12.95	Os08g0110000	None	None
15	RM339	8	17.94	0.0249	7.84	None	None	None
16	RM316	9	1.07	0.0227	12.29	None	None	None
17	RM342B	9	8.55	0.0132	9.26	None	None	None
18	PSM340	9	21.67	0.0217	5.56	None	None	None
19	RM239	10	9.69	0.0229	12.18	None	None	None
20	PSM166	10	14.72	0.0306	9.48	None	Homeobox-leucine zipper protein HOX9	None
21	RM258	10	18.08	0.0489	11.87	Os10g0480200	None	*qALRR-10* (Nguyen et al., 2002)
						Hypothetical conserved gene	
22	PSM365	11	21.39	0.0029	20.03	None		None
23	RM179	12	14.45	0.0156	8.89	Os12g0438400		None

**R*^2^% represents the percentage of phenotypic variance explained by the relevant marker.*

### Performance of Al Tolerance Relevant to Different Alleles of Significant Loci

Six markers, i.e., PSM41, RM7, PSM377, RM252, PSM142, and PSM365, were selected for analysis of Al tolerance performance relevant to different alleles of significant loci based on their high explained phenotypic variation (≥15%, **Table 2**). Six alleles, ranging from 140 to 180 bp, were detected at a locus PSM41 in Ting’s core collection, and the individuals carrying the 180 bp allele (size of PCR product for the SSR markers), i.e., nine varieties including seven typical *japonica* and two *indica* varieties, had a significantly (*P* < 0.05) larger RRE than those carrying other alleles. Four alleles, ranging from 89 to 117 bp, were detected at a locus PSM377, and the individuals carrying the allele 93 bp, i.e., 26 varieties including 17 typical *indica*, five *indica*-clined and four typical *japonica* varieties, had a significantly (*P* < 0.05) larger RRE than those carrying 89 bp allele. Five alleles, ranging from 163 to 201 bp at a locus RM252, were detected, and the individuals carrying the allele of 201 bp had a significantly (*P* < 0.05) smaller RRE than those carrying other alleles of 163, 179, and 198 bp. In addition, a total of four, six, and five alleles were detected in Ting’s core collection with RM7, PSM365, and PSM142, respectively. However, we detected non-significant RRE index for the individuals relevant to different alleles of these loci (**Table 3**).

**Table 3:** Duncan’s test of the Al tolerant performance of rice landraces harboring different alleles of the markers significantly associated with Al tolerance.

Locus	Allele (bp)	Mean ±*SD*	Locus	Allele (bp)	Mean ±*SD*
PSM41	140	0.60 ± 0.18^ACab^	PSM365	132	0.55 ± 0.13^Aa^
	145	0.51 ± 0.14^ABab^		191	0.58 ± 0.17^Aa^
	152	0.53 ± 0.12^ABab^		199	0.52 ± 0.12^Aa^
	167	0.54 ± 0.10^ABab^		221	0.53 ± 0.10^Aa^
	177	0.46 ± 0.12^Ba^		248	0.55 ± 0.14^Aa^
	180	0.65 ± 0.13^Cb^		277	0.52 ± 0.15^Aa^
RM7	139	0.53 ± 0.14^Aa^	PSM377	89	0.51 ± 0.14^Aa^
	152	0.53 ± 0.11^Aa^		93	0.59 ± 0.10^Ba^
	155	0.55 ± 0.14^Aa^		111	0.56 ± 0.18^ABa^
	160	0.55 ± 0.12^Aa^		117	0.53 ± 0.10^ABa^
RM252	163	0.55 ± 0.15^ABab^	PSM142	147	0.52 ± 0.10^Aa^
	166	0.44 ± 0.08^ACab^		156	0.50 ± 0.12^Aa^
	179	0.5 ± 0.08^Ba^		168	0.56 ± 0.16^Aa^
	198	0.53 ± 0.12^ABab^		179	0.57 ± 0.13^Aa^
	201	0.40 ± 0.09^Cb^		184	0.53 ± 0.14^Aa^

*Upper and lower case letters represent significant differences at α = 0.05 and 0.01, respectively.*

We found six varieties carrying 180 and 93 bp alleles at PSM41 and PSM377 loci, and eight varieties exhibited 179 and 93 bp alleles at RM252 and PSM377 loci, respectively (these accessions are shown in Supplementary Table S1). Moreover, the individuals carrying both 180 bp (PSM41) and 93 bp (PSM377) alleles had a larger RRE (0.60 ± 0.14) than those carrying only 93 bp at PSM377 locus (0.57 ± 0.08). We detected larger RRE (0.62 ± 0.13) in the individuals harboring both 179 bp (RM252) and 93 bp (PSM377) alleles than those carrying a single allele, either 179 bp at locus RM252 (0.54 ± 0.06) or 93 bp at locus PSM377 (0.57 ± 0.08) (Supplementary Table S3).

## Discussion

Most of the previous studies on QTL mapping for Al tolerance were based on the selection of typical Al tolerant and sensitive rice accessions for constructing a segregating population and mapping. Different to previous studies, a core collection consisted of rice landraces was used for association mapping for Al tolerance in the present study. As rice landraces represent an intermediate stage in domestication between wild and elite cultivars (Londo et al., 2006), therefore, it contains high level of genetic diversity as well as potential elite genes for Al tolerance and other agronomic traits for genetic improvement of rice. The mapping population in this study, i.e., Ting’ core collection, were collected from wide regions including the main rice growth countries in the very early stage (1920–1964). Moreover, the core collection was constructed based on 15 quantitative traits and 34 qualitative traits from 2262 accessions of rice landraces of Ting’s collection with an optimal sampling strategy (Li et al., 2011). Consequently, screening for Al tolerance and mapping relevant QTLs within the core collection is of significant importance for the effective utilization of rice landraces.

### Selection of Al Toxicity Concentration

There are several nutrient solutions which were used for rice Al tolerance screening. Among them, Yoshida’s rice solution (Yoshida et al., 1976) is a classic one, which had been widely used to Al tolerance screening. However, high concentrations of mineral ions interacting with Al^3+^ in Yoshida’s solution could obviously reduce the activity of Al^3+^ (Famoso et al., 2010). To avoid this, Famoso et al. (2010) modified the Magnavaca’s nutrient solution by using Fe-hydroxyethyl-ethylenediaminetriacetic acid (Fe-HEDTA) chelate to prevent Fe precipitation and citrate interaction with Al, which could greatly maintain the activity of Al^3+^ and can be used to screen Al tolerance in plants at all stages of development (Famoso et al., 2011). A simple CaCl_2_ solution method was also used to screen Al tolerance, especially for rice young seedlings, because at the early stage of development the seed is still capable of providing all necessary mineral nutrients (Ma et al., 2002). This method can effectively avoid the problem of Al precipitation and allows for reproducible Al^3+^ concentrations. Moreover, Xue et al. (2005) compared the two Al tolerance screening methods, i.e., the complete nutrient solution and the simple CaCl_2_ solution, to detect Al tolerance of two rice varieties Asominori (Al tolerant) and IR24 (Al sensitive) and found that the two screening methods showed non-significant difference. Therefore, as we used young seedlings to screen Al tolerance in this study, the simple CaCl_2_ solution method was applied.

In the previous studies, concentration for Al toxicity solutions was selected in a wide range due to different container, nutrient solution, treatment duration and other experimental factors. For instance, 20, 30, 160, and 160 μmol l^-1^ of AlCl_3_ were selected by Huang et al. (2009), Famoso et al. (2010), Xia et al. (2010), and Famoso et al. (2011), respectively. In this study, five tolerant and sensitive rice varieties were used to determine 100 μmol l^-1^ of AlCl_3_ as an appropriate concentration for Al toxicity. Similarly, Fu et al. (2010) found that the 100 μmol l^-1^ of AlCl_3_ can differentiate between Al tolerant and sensitive varieties by using RRE ≥ 0.5 as a criterion for Al tolerant variety, and many rice varieties had been identified as tolerant or sensitive varieties using this criterion. This concentration was also used in the research of Xue et al. (2007), Zhang et al. (2007), and Cai et al. (2012). Therefore, the concentration for estimating the Al toxicity in this study, combined with RRE ≥ 0.5 as Al tolerance level, is a reliable approach to measure the degree of Al tolerance in rice landraces.

### Performance of Al Tolerance in Ting’s Core Collection

As expected, our results indicated that there was a wide-range of phenotypic variation for Al tolerance in Ting’s core collection. More than 63.70% varieties in Ting’s core collection were found to be Al tolerant, which could be used as donor parents to introgress Al tolerance in elite rice varieties. We identified both *indica* and *japonica* rice varieties as Al tolerant varieties. Our results are in fully agreement with the Famoso et al. (2011), who also found Al tolerant varieties both in *indica* and *japonica* rice.

As 42.0% of phenotypic variation of Al tolerance was influenced by population structure, it is necessary to compare the Al tolerance within subgroups and even sub-subgroups. Both the percentage of Al tolerant varieties and mean of RRE in SG 2 were the largest (**Figure 3**; **Table 2**), which suggested that *japonica* might have more Al tolerant varieties than *indica* and admixed groups (mixture of *indica* and *japonica*). These findings were fully consistent with the study of Famoso et al. (2011). Moreover, the percentages of Al tolerant varieties and mean of RRE in AD were higher than other subgroups and sub-subgroups except SG 2 (**Figure 3**; **Table 2**), which might be attributed to the more number of *japonica* varieties in AD (four typical *japonica* rice, eight *japonica*-clined rice, and seven *indica*-clined rice). The percentage of Al tolerant varieties and mean of RRE in SG 1b were the smallest, which suggested that the rice varieties from subtropical region are more sensitive to Al toxicity than other regions, and required the introgression of Al tolerance genes from other regions to produce resistance in these varieties.

### Association Analysis for Al Tolerance in Ting’s Core Collection

We used the MLM (Q+K; Yu et al., 2006) approach, which accounted for population structure and kinship relationship to minimize spurious associations, to perform association mapping for Al tolerance in Ting’s core collection, which was genotyped by using 274 SSR markers. Our previous research indicated that LD decayed to 75% quantile of *r*^2^ for unlinked loci at 40–50 cM (Zhang et al., 2011), which was similar to the research of Jin et al. (2010) where LD did not decay until 25–50 cM in a set of germplasm consisting of 416 rice accessions assessed with 100 SSR markers. Moreover, the LD blocks observed in our previous study had an average length of 7.1 cM, and the number of LD blocks for the core collection was 56. Therefore, though the number of SSR markers used in this study was low compared to the genome-wide high density single nucleotide polymorphisms (SNPs), association mapping with relative low number of markers is still possible. Such explanation could be supported by previous researches (e.g., Stich et al., 2008; Stich and Melchinger, 2009).

We detected significant (*P* < 0.05) trait–marker associations for Al tolerance on all rice chromosomes, while few rice chromosomes were found to harbor QTLs in previous studies. For instance, Famoso et al. (2011) detected a total of 3, 1, 1, 1, and 3 QTLs on chromosome 1, 2, 6, 9, and 12 by linkage mapping. Moreover, they cannot found any significant association on chromosome 8 by association mapping. We used a core collection of rice landraces, while two bi-parental segregation mapping populations and a rice panel consisting of 383 diverse rice accessions, which was not developed as a core collection, were used in the study of Famoso et al. (2011), thus different mapping populations might be the reason for different results in both studies. We detected about 13% (three loci, **Table 2**) significant (*P* < 0.05) trait–marker associations identical to the QTLs reported previously, i.e., RM7 is located in the region of QTL *CQL3* (Wu et al., 2000), PSM142 is located in the vicinity of QTL *QAlRr7.1* (Nguyen et al., 2003), and RM258 was found to be in the region of QTL *qALRR-10* (Nguyen et al., 2002). The other significant associations found in this study, might be new novel loci for Al tolerance. For example, we detected nine significant associations located in the vicinity of candidate genes, i.e., PSM41 is located in the flanking region of *Os01g0140700*, which is a regulator of the ATPase of the vacuolar membrane; RM156 is located in the vicinity of *Os03g0424500*, which encodes a 40S ribosomal protein S193; RM136 is located in the region of *Os06g0264800*, which encodes a protein CURVATURE THYLAKOID 1A, chloroplastic; RM407 was found to be in the flanking region of *Os08g0110000*, which encodes an alpha/beta hydrolase fold-1 domain containing protein; RM258 is located in the region of *Os10g0480200*, which is similar to rolled leaf1 and homeobox-leucine zipper protein HOX9; RM220, RM341, RM179, and PSM377 found to be in the regions of *Os01g0183633, Os02g0527900*, which have been annotated as protein(s) or gene(s) associated with important biological processes, respectively.

In addition, we found four loci that were located certain distance away from the regions containing genes or QTLs reported by previous studies. There was a 0.35 Mb genetic distance between RM220 and *OsCDT3* (encoding a plasma membrane-localized small peptide; Xia et al., 2013). PSM377 located at a distance of 3.8 cM from *CQL3* (Wu et al., 2000), while RM156 was located at a distance of 6.5 cM from *qALRR-3* (Nguyen et al., 2002). RM407 and *QA1Rr8.1* (Nguyen et al., 2003) was 3.4 cM apart from each other. There is a possibility of QTLs *DTH12* and *Hd13* are being the same (detected in different mapping populations), though they have found a distance of 2.5 Mb between two QTLs (Zhong et al., 2014). Further studies, especially map-based cloning and sequence analysis, are needed to confirm the linkage between these loci and previous QTL(s) or gene(s).

Based on our mapping results, the significant (*P* < 0.05) trait–marker associations with high *R*^2^ deserve to study further. Our results indicated that the alleles with 180 bp of PSM41, 179 bp of RM252, and 93 bp of PSM377 might be the superior alleles for Al tolerance. Interestingly, 77.80% of the nine varieties harboring 180 bp allele at locus PSM41 were typical *japonica*, 96.00% of the 25 varieties carrying 179 bp allele at locus RM252 were *indica*, and 80.80% of the 26 varieties containing 93 bp allele at locus PSM377 were *indica*. Both *indica* and *japonica* cultivars showed remarkable differences for Al tolerance mechanism during evolution (Famoso et al., 2011). Furthermore, the six varieties carrying the alleles of 180 and 93 bp as well as the eight varieties carrying the alleles of 179 and 93 bp are worthy of further studies. These elite alleles for Al tolerance could be used for developing rice cultivars with Al tolerance through marker-assisted breeding. Taken together, these results can be used as a reference for molecular breeding to improve Al tolerance in rice.

## Author Contributions

PZ and JL conceived and designed the experiments. PZ performed the experiments. PZ, JL, KZ, and HT analyzed the data. JL, PZ, and MS contributed reagents/materials/analysis tools. PZ, JL, KZ, and MS wrote and revised the manuscript.

## Conflict of Interest Statement

The authors declare that the research was conducted in the absence of any commercial or financial relationships that could be construed as a potential conflict of interest.

## Footnotes

1 http://ibi.zju.edu.cn/software/qga/

2 http://www.maizegenetics.net/tassel

## Abbreviations

Al^3+^: trivalent aluminum
GLM: general linear model
MLM: mixed linear model
PIC: polymorphism information content
QTL: quantitative trait locus
RRE: relative root elongation
SD: standard deviation
SSR: simple sequence repeat
