# Supplementary material for: Association Mapping for Aluminum Tolerance in a Core Collection of Rice Landraces
Source: Front Plant Sci. 2016 Oct 4;7:1415. doi: 10.3389/fpls.2016.01415 (PMC5047912; doi:10.3389/fpls.2016.01415)
Supplement: Supplementary file 1 [file Table_1.DOCX]

Table S1 Accessions, variety names, origin, germplasm types and RRE of 150 rice varieties in Ting’s core collection*

| Acc. | Variety name | Origin | *Indica* vs.  *Japonica* | Relative root elongation (RRE)±S.D. | Acc. | Variety name | Origin | *Indica* vs. *Japonica* | Relative root elongation (RRE)±S.D. |
| --- | --- | --- | --- | --- | --- | --- | --- | --- | --- |
| CC1 | Yin guang | Japan | *TJ* | 0.77±0.03 | CC76^￥^ | Guang ye hong mi | South China | *TI* | 0.51±0.02 |
| CC2 | Ao guo 5-B | Japan | *TJ* | 0.68±0.32 | CC77^￥^ | Da nuo | South China | *TI* | 0.66±0.02 |
| CC3 | Ai you | Japan | *TJ* | 0.89±0.16 | CC78^※^ | Bai xu | South China | *TI* | 0.53±0.06 |
| CC4 | Tie geng yi shi ao | Yangtze River region | *TJ* | 0.29±0.01 | CC79 | Mao he | South China | *TI* | 0.47±0.15 |
| CC5 | Guo zhu | Japan | *TJ* | Missing | CC80 | Xu zai | South China | *TI* | 0.38±0.04 |
| CC6^＃^ | Ben dao | North China | *TJ* | 0.86±0.18 | CC81 | Dong an hou zi pu xiao he | Central China | *TI* | 0.39±0.05 |
| CC7^＃^ | Mang shui dao | Yangtze River region | *TJ* | 0.50±0.03 | CC82 | Tie gu pao | Central China | *TI* | 0.32±0.08 |
| CC8 | Bai mang gao li han dao bai | North China | *TJ* | 0.57±0.37 | CC83^￥※^ | Chi mao zhan | South China | *TI* | 0.51±0.03 |
| CC9^＃※^ | Jiu yue han | Northeast China | *TJ* | 0.52±0.13 | CC84 | Hu bei zao | Central China | *TI* | 0.59±0.04 |
| CC10^＃※^ | Bi jie ma wei hong gu | Yunnan-Kweichow Plateau | *TJ* | 0.84±0.01 | CC85^￥^ | Ya jing mi | South China | *TI* | 0.51±0.25 |
| CC11 | Ai da tou | Yangtze River region | *TJ* | 0.50±0.11 | CC86 | Ba shi zi | Central China | *TI* | 0.71±0.10 |
| CC12 | Gui zao bai he | Yangtze River region | *TJ* | 0.43±0.02 | CC87^￥^ | Dong jun zi | Central China | *TI* | Missing |
| CC13^＃^ | Xiang dao | North China | *TJ* | 0.52±0.31 | CC88^￥^ | Early | Unknown | *TI* | 0.54±0.08 |
| CC14^＃※^ | Zi jin gu | Northeast China | *TJ* | 0.61±0.33 | CC89 | Nuo | South China | *TI* | 0.71±0.16 |
| CC15^※^ | Xiang chuan | Japan | *TJ* | 0.62±0.23 | CC90 | Gui zhao he 2 | Japan | *TI* | Missing |
| CC16 | Nagabo | Taiwan | *TJ* | 0.51±0.05 | CC91 | Hei nuo | Unknown | *TI* | 0.37±0.04 |
| CC17 | Bai ke da nuo | South China | *TJ* | 0.47±0.11 | CC92 | Da yi mao | Central China | *TI* | 0.49±0.17 |
| CC18 | San pai zhong | South China | *TJ* | 0.50±0.01 | CC93 | Gai cao zhan | Central China | *TI* | 0.50±0.01 |
| CC19 | Kai xuan | Japan | *TJ* | 0.30±0.09 | CC94 | Gamal | Unknown | *TI* | 0.56±0.10 |
| CC20 | Shi ban zhan | North China | *TJ* | 0.86±0.08 | CC95^※^ | Bu gou wei | South China | *TI* | 0.64±0.14 |
| CC21 | Hei ke da nuo | South China | *TJ* | 0.30±0.00 | CC96 | Bai ke xi nuo | South China | *TI* | 0.53±0.21 |
| CC22 | Shen shui wan dao | Yangtze River region | *TJ* | 0.64±0.21 | CC97^￥※^ | Ben dao | North China | *TI* | 0.61±0.20 |
| CC23 | Hong ben dao | Yangtze River region | *JC* | 0.59±0.05 | CC98 | Ba xian shu | Japan | *TI* | 0.67±0.20 |
| CC24^＃※^ | Duan mang zi jin gu | Northeast China | *TJ* | 0.56±0.12 | CC99 | Guang hong mi dao | Yangtze River region | *TI* | 0.54±0.13 |
| CC25 | Bei jing jiang mi | North China | *JC* | 0.68±0.11 | CC100 | Wu mang yan guo qing | North China | *TI* | 0.34±0.03 |
| CC26 | Daeri | Celebes | *JC* | 0.56±0.07 | CC101^※^ | Chang xu nuo | South China | *TI* | 0.53±0.05 |
| CC27^￥^ | Jian tou nuo | South China | *JC* | 0.60±0.02 | CC102 | Jiang wan 15 | Central China | *IC* | 0.65±0.25 |
| CC28 | Long you man dao | Yangtze River region | *TJ* | 0.37±0.01 | CC103^※^ | Bai hua er | South China | *TI* | 0.58±0.07 |
| CC29 | Kun shan zhu zhou dao | Yangtze River region | *JC* | 0.77±0.00 | CC104^￥^ | Liu chang xian | South China | *TI* | 0.50±0.10 |
| CC30 | Huang ke zao 2 | Yangtze River region | *JC* | 0.51±0.06 | CC105^￥^ | Bai yin 3 | South China | *TI* | 0.52±0.13 |
| CC31 | Sheng fang da bai gu | North China | *JC* | 0.68±0.04 | CC106^￥※^ | Shui zao huang pi | South China | *TI* | 0.53±0.31 |
| CC32 | Xiao dou | Japan | *JC* | 0.59±0.08 | CC107 | Yin 2 dong 7 | South China | *TI* | 0.64±0.10 |
| CC33^＃※^ | Poetih | Celebes | *IC* | 0.71±0.13 | CC108^￥^ | Hou ma | South China | *TI* | 0.50±0.11 |
| CC34^＃※^ | Tebaro | Sumbawa | *IC* | 0.51±0.28 | CC109 | Dong zhu 2 | South China | *TI* | 0.59±0.21 |
| CC35 | Ao hua da gui tou hong | Yangtze River region | *IC* | 0.57±0.09 | CC110 | Hong gen da mi | South China | *TI* | 0.37±0.04 |
| CC36 | Hui bei zi | Yunnan-Kweichow Plateau | *IC* | 0.47±0.08 | CC111 | Ben cheng guan yin zhan | Central China | *TI* | 0.55±0.14 |
| CC37 | Ba shi zi | Yangtze River region | *IC* | 0.60±0.01 | CC112^￥※^ | Xi miao gu | South China | *IC* | 0.56±0.06 |
| CC38 | Zao sheng da ye | Japan | *IC* | 0.47±0.01 | CC113^※^ | 186-zao guan yin zhan | Central China | *TI* | 0.63±0.01 |
| CC39 | Bnlastog | Low latitude region | *IC* | 0.64±0.11 | CC114 | Chang mang hei ma zao | Yunnan | *TI* | 0.54±0.10 |
| CC40^￥^ | Nuo mi | North China | *IC* | 0.50±0.21 | CC115 | Shui tian zhan gu nuo | South China | *TI* | 0.49±0.00 |
| CC41 | Xi chuan huang liu | South China | *TI* | 0.49±0.06 | CC116 | Chang han da hua ke | South China | *IC* | 0.48±0.07 |
| CC42 | Hei ju dao | Yangtze River region | *TI* | 0.43±0.16 | CC117^￥※^ | Da he | South China | *TI* | 0.68±0.17 |
| CC43^※^ | Guang fuⅠ | Taiwan | *TI* | 0.45±0.01 | CC118^※^ | Zeng cheng hei nuo | South China | *TI* | 0.77±0.15 |
| CC44 | Zhong qi jia qing | Yangtze River region | *IC* | 0.43±0.07 | CC119 | Mao he | South China | *TI* | 0.48±0.14 |
| CC45 | Ⅲ-49-4xi chuan huang | Taiwan | *IC* | 0.46±0.27 | CC120 | Bai gu zhan | Central China | *TI* | 0.33±0.19 |
| CC46^￥※^ | Xin xian li | Yangtze River region | *TI* | 0.75±0.07 | CC121 | Die zhi | South China | *TI* | 0.49±0.13 |
| CC47^￥^ | Da liu tiao dao | Yangtze River region | *IC* | 0.62±0.00 | CC122 | You zhan | South China | *TI* | 0.47±0.04 |
| CC48 | Bai ke | South China | *TI* | 0.66±0.26 | CC123 | Chang sha wu qu wan dao | Central China | *TI* | 0.51±0.06 |
| CC49 | Chuan chi 1 | Central China | *TI* | 0.33±0.01 | CC124 | Tong ling hu nan xian | Central China | *TI* | 0.36±0.21 |
| CC50 | Tai nong 46 | Taiwan | *TI* | 0.44±0.05 | CC125 | Xiao mao dao | Central China | *TI* | 0.59±0.06 |
| CC51 | Ba chong sui | Japan | *TI* | 0.45±0.21 | CC126 | Jing xian si qu er gan | Central China | *TI* | 0.48±0.12 |
| CC52 | Yun nan bai | Central China | *TI* | 0.60±0.01 | CC127 | Zi xing er qu si dou xu | Central China | *TI* | 0.52±0.10 |
| CC53 | Liao yang ben di 4 | Northeast China | *IC* | 0.65±0.37 | CC128 | Chen hui fu dao | Central China | *TI* | 0.28±0.09 |
| CC54 | You zhan hong | South China | *TI* | 0.41±0.11 | CC129 | Bai gan zi | Central China | *TI* | 0.45±0.23 |
| CC55 | Hei nuo | South China | *TI* | 0.28±0.06 | CC130 | Han lu wei zhan | Central China | *TI* | 0.61±0.17 |
| CC56 | Xian zi zhan | Central China | *TI* | 0.42±0.21 | CC131 | Xin hua san qu tang mao zhan | Central China | *TI* | 0.46±0.08 |
| CC57 | Da tou meng | Central China | *IC* | 0.72±0.23 | CC132 | Ta gu zhan | Central China | *TI* | 0.51±0.02 |
| CC58 | Chi bai gan zhan | Central China | *TI* | 0.35±0.22 | CC133 | Mian tiao zhan | Central China | *TI* | 0.39±0.04 |
| CC59^※^ | Zeng cheng xiang shan zhan | South China | *TI* | 0.54±0.16 | CC134 | Hu guang zhan | Central China | *TI* | 0.49±0.05 |
| CC60^￥^ | Cang wu shan he zhan | South China | *TI* | 0.52±0.20 | CC135 | Jiang an da ye zao | Central China | *TI* | 0.47±0.05 |
| CC61 | Da gu zao | South China | *TI* | 0.55±0.04 | CC136 | Tie ban zhan | Central China | *TI* | Missing |
| CC62 | Jie yang dong liao zhong | South China | *TI* | 0.72±0.20 | CC137 | Da gu zao | Central China | *TI* | 0.45±0.06 |
| CC63^￥^ | Nan xiong ku gua zao | South China | *TI* | 0.53±0.02 | CC138 | Ding nan dong zhan | Central China | *TI* | 0.49±0.06 |
| CC64 | Chang mang | South China | *TI* | 0.68±0.36 | CC139 | Lao wu gu | Central China | *TI* | 0.63±0.00 |
| CC65^※^ | Mandi | Celebes | *TI* | 0.46±0.02 | CC140 | Xin ban chang ke zi | Central China | *TI* | 0.64±0.15 |
| CC66 | Bai gu | South China | *TI* | 0.42±0.04 | CC141^※^ | Bai zhan gu | Central China | *TI* | 0.56±0.19 |
| CC67 | Hong zao gu | Yunnan-Kweichow Plateau | *TI* | 0.59±0.04 | CC142^￥※^ | Si chuan zhan | Central China | *TI* | 0.54±0.01 |
| CC68 | Cang wu shan he zhan | South China | *TI* | 0.54±0.01 | CC143 | Gao jiao gui hua | Central China | *TI* | 0.54±0.01 |
| CC69 | Zao die zhan gu | Central China | *TI* | 0.45±0.17 | CC144 | Chang shu wu wi dao | Central China | *TI* | 0.49±0.08 |
| CC70 | Su zhou zhan | Central China | *TI* | 0.58±0.11 | CC145^※^ | Da nuo bai dong | Central China | *TI* | 0.57±0.16 |
| CC71 | Yang zhan 3 | South China | *TI* | 0.50±0.00 | CC146^￥※^ | Lin chuan da ye zao | South China | *TI* | 0.66±0.02 |
| CC72 | Luo ding zhan 1 | South China | *TI* | 0.54±0.29 | CC147^￥^ | Da bai cao | North China | *TI* | 0.51±0.21 |
| CC73^￥^ | Gen yin 29 | South China | *TI* | 0.71±0.32 | CC148 | Chang ning wu qu nan tou zhan | Central China | *TI* | 0.22±0.03 |
| CC74^￥^ | Wu ke nuo | South China | *TI* | 0.44±0.08 | CC149 | Pi xian da ye zi | Central China | *TI* | 0.41±0.12 |
| CC75 | Hua bai ke | South China | *TI* | 0.44±0.01 | CC150^￥^ | Xi zi zhan | Central China | *TI* | 0.58±0.22 |

* *Indica* or *japonica* characteristic were identified by Cheng’s index, i.e. TI, typical *indica*, IC, *indica*_clined, JC, *japonica*_clined, and TJ, typical *japonica*. ＃represents the varieties which were genotyped as allele 180bp (PSM41), ￥represents the varieties which were genotyped as allele 179bp (RM252), ※represents the varieties which were genotyped as allele 93bp (PSM377).
