# Supplementary material for: Association Mapping for Aluminum Tolerance in a Core Collection of Rice Landraces
Source: Front Plant Sci. 2016 Oct 4;7:1415. doi: 10.3389/fpls.2016.01415 (PMC5047912; doi:10.3389/fpls.2016.01415)
Supplement: Supplementary file 3 [file Table_3.DOCX]

Table S3 Duncan test for pyramiding effect of superior alleles for Al tolerance

| Allele (bp) | Mean ±S.D. |
| --- | --- |
| 180 at locus PSM41 and 93 at locus PSM377 | 0.60±0.14 ^Aa^ |
| 179 at locus RM252 and 93 at locus PSM377 | 0.62±0.13 ^Aa^ |
| 180 at locus PSM41 | 0.62±0.20 ^Aa^ |
| 179 at locus RM252 | 0.54±0.06 ^Aa^ |
| 93 at locus PSM377 | 0.57±0.08 ^Aa^ |

Note: Capital and lower letter represented significant correlation at α=0.05 and 0.01, respectively.
